# Dairy cows welfare quality in tie-stall housing system with or without access to exercise

**DOI:** 10.1186/1751-0147-55-43

**Published:** 2013-06-01

**Authors:** Silvana Popescu, Cristin Borda, Eva Andrea Diugan, Marina Spinu, Ioan Stefan Groza, Carmen Dana Sandru

**Affiliations:** 1 Department of Animal Hygiene and Welfare, Faculty of Veterinary Medicine, University of Agricultural Sciences and Veterinary Medicine, Manastur Street 3-5, 400372 Cluj-Napoca, Romania; 2 Department of Infectious Diseases and Preventive Medicine, Faculty of Veterinary Medicine, University of Agricultural Sciences and Veterinary Medicine, Manastur Street 3-5, 400372 Cluj Napoca, Romania; 3 Department of Animal Reproduction, Obstetrics and Reproductive Pathology, Faculty of Veterinary Medicine, University of Agricultural Sciences and Veterinary Medicine, Manastur Street 3-5, 400372 Cluj Napoca, Romania

**Keywords:** Dairy cow, Welfare quality, Tie-stall housing, Animal based measure

## Abstract

**Background:**

Tie-stall housing of dairy cows is used extensively worldwide, despite of the welfare concerns regarding the restriction of voluntary movement and limitation of expression of the cows’ natural behaviour. The aim of this study was to compare the welfare quality of dairy cows kept in two types of tie-stall housing systems: with regular outdoor exercise and without access to exercise. In addition, the study investigated the relationship between different welfare measures of dairy cows kept in tie-stalls.

**Methods:**

3,192 lactating cows were assessed using the Welfare Quality® assessment protocol for cattle in 80 commercial dairy farms, half of the farms providing outdoor access for the animals to exercise. The descriptive statistical indicators were determined for the assessed measures and for the welfare criteria and principle scores. The data obtained in the two housing types were compared and the correlation coefficients were calculated between the different welfare measures.

**Results:**

The significant differences found between the two housing systems for the majority of the animal based measures indicate the positive effect of exercise on the welfare of tethered cows. Many of the animal welfare parameters correlated with each other. For the farms allowing the cows’ turnout in a paddock, pasture or both, the mean scores for the welfare criteria and principles were higher than for the farms with permanent tethering of the cows, except the criteria absence of prolonged hunger and expression of social behaviours. The lowest scores were obtained for the criterion positive emotional state, in both housing systems. With regard to the overall classification, none of the farms were considered excellent. In the not classified category were only farms with all-year-round tethering of the animals and in the enhanced category only farms where the cows had outdoor access.

**Conclusions:**

The welfare quality of the investigated dairy cows was significantly better in the tie-stall farms which allow exercise for cows (paddocks, pasture or both) than in those which do not. In the light of our results we consider that dairy cattle welfare is not necessarily poor in tie-stall housing systems, its quality depending on the management practices.

## Background

Despite growing criticism, tie-stall housing systems are still extensively used for dairy cows in many parts of the world. In Europe, between 20% (lowland) and 80% (upland) of cows are tethered at least during the winter [1]. Research papers show that approximately 88% of Norwegian dairy cattle [2], 75% of all Swedish dairy herds [3] and more than one third of German dairy cows [4] are kept in tie-stall housing systems, often without pasturing. According to the 2007 USDA report [5] 62% of US dairy farms had tie-stall barns. In Romania, the tied system is used in approximately 75% of the middle sized and large farms and in 90% of the small farms (e.g. less than 30 cows) (personal observation, Popescu). The decision of farmers to keep dairy cows tethered is motivated in the first place by economical reasons, lack of space, equipment and sometimes also by convenience.

In terms of animal welfare, the tie-stall housing system of dairy cows is controversial. According to some authors this system is unacceptable because it restricts the voluntary movement possibilities and the social behaviour of dairy cows [6]. Yet, when periods of exercise are possible, some of the adverse effects are reduced [7-9]. Loberg et al. [3] describe that the longer the cows are tethered in the barns, the more active they are in the paddocks, suggesting a rebound effect (compensatory increase) on locomotion and maybe also on exploratory behaviour. Additionally, Veissier et al. [1] did not find any acute or chronic physiological stress response in cows kept in a tethered housing system.

Lately many studies of comparative research (tie vs. free stalls) focused especially on the health measures but there are only a few studies [9-12] on the welfare of tie-stall housed dairy cows (assessed by different methods) and the results are inconsistent. Regula et al. [9] and Seo et al. [10] suggest that the welfare is better if the tethered cows benefit from regular exercise outdoors. When comparing the welfare parameters of dairy cows allowed to pasture and of those kept tethered all year round Vučemilo et al. [12] report in a recent study that especially for the behavioural indicators the differences are significant.

The aim of this study was to compare the welfare quality of dairy cows kept in two types of tie-stall housing systems: with regular outdoor exercise (paddock or pasture or both) and without access to exercise. In addition, the study investigated the relationship between different welfare measures of dairy cows kept in tie-stall housing systems. The importance of this study and further research of this type lies in the impact it would have on labelling permanent tethering of dairy cattle as unacceptable on animal welfare considerations. If there were stronger scientific arguments that outdoor access of the cows can improve their welfare, there would be more opportunities at country or European level (official recommendations and guidelines, project financing to give incentive to farmers, legal decisions of veterinary and agricultural authorities) to proceed towards abolishing permanent tethering of dairy cattle.

## Methods

### Study design

The study was completed in 80 commercial dairy cattle farms in Transylvania, Romania, selected to fulfil the following criteria: tie-stall housing system with access to exercise (THSE) and without access to exercise (THSNE), minimum 30 cows, milk delivery to processing units, easy access to the farm in winter conditions, and the cost-free availability of the farmer to participate to the study. The main characteristics of the farms were as follows: all the cattle barns were closed by entire walls; the number of the cows/farm varied between 30 and 113 (69.18 ± 3.41 lactating cows, mean ± SE); the breeds were Holstein (25%) and Romanian Spotted cattle (75%); the milk yield per cow per day was 16.73 ± 0.41 (mean ± SE) kg; the cows were kept on stalls with lengths between 160 cm and 250 cm, and widths between 85 cm and 190 cm; bedding (straw, sawdust) was used in all of the barns but generally in small quantity (1.5 kg/head/day or less). Manure cleaning of the barns was made manually (in 40% of the farms) or mechanically (in 60% of the farms). Cows were milked in their stalls twice daily at 06.00 and 18.00 h. Only in half of the selected farms (n = 40) the cows had access to free outdoor exercise (paddock, pasture or both). The cows were pasturing on average 10.65 hours a day, 182 days a year. The study was accomplished in the cold period of the year, when the cows were housed (November – February).

### Welfare assessment

The cows’ welfare assessment on selected farms was done by applying the Welfare Quality® Assessment Protocol for Cattle [13], which include four major welfare principles, 12 criteria and 29 measures (Table 1). The majority of the measures were recorded directly at animal level; a few parameters regarding the resources and management of the farm such as access to outdoor loafing area or pasture, mortality, dystocia, downer cows, disbudding/dehorning and tail docking were provided by the farmer. A total number of 3,192 milked cows were assessed; their number in each farm was established according to the instructions of the assessment protocol. In those farms where not all the animals were included in the study, the cows were selected randomly at the beginning (during milking), marked with an animal marker on their withers and assessed in accordance with all the instructions of the protocol. Data collection was done in each farm in the morning, after milking. The assessment methods used were observation, clinical exam, measuring, chronometry, questioning of the farmers and calculations.

**Table 1:** **The principles**, **criteria and measures of the Welfare Quality**® **assessment protocol for dairy cows**

**Welfare principles**	**Welfare criteria**	**Assessed measures**
1. Good feeding	1. Absence of prolonged hunger (APH)	Body condition score
	2. Absence of prolonged thirst (APT)	Water provision; cleanliness of water points; water flow; functioning of water points
2. Good housing	3. Comfort around resting (CAR)	Time needed to lay down; animals colliding with housing equipment during lying down; animals lying partly or completely outside the lying area; cleanliness of udders, flank/upper legs, lower legs
	4. Thermal comfort	As yet, no measure is developed. For the study the momentary temperature was recorded
	5. Ease of movement (EM)	Presence of tethering; access to outdoor loafing area or pasture
3. Good health	6. Absence of injuries (AI)	Lameness; integument alterations
	7. Absence of disease (AD)	Coughing; nasal discharge; ocular discharge; hampered respiration; diarrhoea; vulvar discharge; milk somatic cell count; mortality; dystocia; downer cows
	8. Absence of pain induced by management procedures (APIMP)	Disbudding/dehorning; tail docking
4. Appropriate behaviour	9. Expression of social behaviours (ESB)	Agonistic behaviours – assessed by observation of head butts; displacements; chasing; fighting; chasing-up
	10. Expression of other behaviours (EOB)	Access to pasture
	11. Good human-animal relationship (GHAR)	Avoidance distance
	12. Positive emotional state (PES)	Qualitative behaviour assessment – by observation of the cows’ 'body language' regarding 20 behavioural terms (active, relaxed, fearful, agitated, calm, content, indifferent, frustrated, friendly, bored, playful, positively occupied, lively, inquisitive, irritable, uneasy, sociable, apathetic, happy, distressed)

In each farm the cows were evaluated once, by two assessors trained previously in several different dairy farms until at least 80% intra- and interassessor reliability was reached. Data recorded on the farms were processed using the software program of the Welfare Quality® scoring system [14], computing the criterion and principle scores and finally classifying the farms in a welfare category: not classified, acceptable, enhanced or excellent [13]. More detailed information regarding data collection, calculation and practical meaning of the scores and classification of the farms can be found in the Welfare Quality® assessment protocol for dairy cows [13].

### Statistical analysis

The descriptive statistical indicators (mean, standard error of the mean, median, minimum and maximum) were determined for the assessed measures and for the scores of the 11 criteria and four welfare principles (Table 1). The comparison of data obtained in the two different housing types was conducted using the t test or the Mann-Whitney test, depending on the normal or abnormal distribution of the data, established with the Kolmogorov - Smirnov Test. The correlation of different welfare measures was described with the Pearson (r_p_) or Spearman rank (r_s_) correlation coefficient, depending on data distribution. The P values less than 0.05 were considered to be significant. All statistical analyses were performed using SPSS for Windows version 17 software (SPSS Inc., Chicago, U.S.A.).

## Results and discussion

### Animal based measures of good feeding and housing

The results obtained for the animal based measures related to the principles of good feeding and housing indicated welfare problems in both housing systems but these were more severe in those farms where the cows did not have access to free exercise. Significant differences were found between the two housing systems for several measures (Table 2).

**Table 2:** **Animal based measures related to the principles of good feeding and housing in tie**-**stall housing system with vs**. **without exercise**

**Measures**	**Tie**-**stall housing system with exercise**				**Tie**-**stall housing system without exercise**			
	**(n = 40)**				**(n = 40)**			
	**Mean**	**SEM**	**Median**	**Range**	**Mean**	**SEM**	**Median**	**Range**
% of very lean cows	9.12	1.60	6.56	32.43	9.49	1.60	8.16	37.14
Duration of lying down movements	5.41	0.09	5.42	2.43	6.77	0.16	6.47^***^	3.16
% lying down movements with collisions	21.07	3.09	17.64	59.18	44.57^***^	4.22	41.00	83.67
% lying cows which lie partly outside lying area	0.78	0.55	0.00	15.62	5.35^***^	1.31	0.00	26.92
% cows with dirty lower legs	31.08	1.15	31.97	29.27	35.09	2.78	31.97	85.72
% cows with dirty udder	25.84	1.14	26.97	28.04	29.04	2.20	28.57	63.06
% cows with dirty flank and upper legs	42.08	1.01	40.00	21.90	43.54	1.93	42.85	55.71

^***^ significant statistical differences at P< 0.001.

n = number of farms.

SEM = standard error of the mean.

The percentage of very thin cows was similar in the two housing systems (Table 2), but higher than recorded in other studies [9,11,15]. In tied housing systems usually the feeding space is properly provided and each cow can consume undisturbed (without competition) the quantity of food supplied. Thus, a possible explanation for the high percentage of very thin cows in this study would be the improper quality and especially quantity of the food offered. However it is possible for the cows to become thin also because of several underlying pathological conditions. For example, in our study a significant correlation of moderate size was found between the percentage of very thin cows and those with diarrhoea (r_s_ = 0.51, P < 0.001) and a weak correlation with those coughing (r_s_ = 0.25, P = 0.03). This measure (very thin cows) correlated positively also with the dirty water bowls (r_s_ = 0.45, P = 0.004) and insufficient water flow (r_s_ = 0.35, P = 0.028). When considering all these aspects and also the mean daily milk-yield, which was rather low in the assessed farms, the effects of water restriction could be taken into account as well. The coping mechanisms of dairy cows to water shortage (by dehydration in the diarrhoeic cows and by insufficient drinking possibility) include the decrease in food intake (a reduction in meal size) and a lower milk production [16].

The mean duration of lying down time was significantly higher in the cows in THSNE farms (U = 110, P < 0.001), but in both systems it exceeded 5.20 seconds which is the value considered normal [13]. In farms with THSNE the mean laying down time exceeded 6.30 seconds, indicating serious welfare problems [13]. These results are in agreement with other studies [8,17] showing that access to exercise is shortening the dairy cows’ lying down duration. However, Loberg et al. [3] did not find any effect of exercise on daily cows’ lying down time. In tie-stalls cows may have problems to lie down due to bad design of the tethers, the manger edge being too high or too short stalls [18,19]. According to Jensen [20] a longer duration of the lying down movement may be caused by the animals’ finding difficult to place the hind legs on the concrete floor when lowering the hindquarters. It may also be that the tether restricts the movement forwards and downwards when kneeling, and this prolongs the movement. The causes leading to increased laying down time include also the painful conditions of the feet, vertebral column and udder [21]. There were strong correlations found between the duration of lying down movements and the percentage of lame cows (r_s_ = 0.79, P < 0.001), the percentage of cows with at least one lesion (r_s_ = 0.56, P < 0.001), the percentage of cows with mastitis (r_s_ = 0.59, P < 0.001) and moderate correlations between the lying down duration and the percentage of cows with at least one hairless patch and no lesion (r_s_ = 0.48, P < 0.001) and the percentage of cows with increased respiratory rate (r_s_ = 0.38, P = 0.001). The motivation of cows to lie down is influenced also by the hygiene of the surfaces and of the bedding, the cows preferring clean, dry and soft surfaces for rest [22]. The cleanliness of the resting surfaces can affect also the time needed to lie down, extending it if the surfaces are covered by manure and are slippery. In line with the results of DeVries et al. [23] we found significant correlations (P < 0.05) between the lying duration and the percentage of cows with dirty upper legs/flank and udder (r_s_ = 0.21, r_s_ = 0.12 respectively).

The frequency of collisions with the housing equipment during lying down (exceeding 20%) represents a welfare problem in both of the housing types [13], being significantly higher (U = 490, P < 0.001) in the THSNE farms (Table 2). The unbalance of the animal when lying down and the consequent hitting of the physical elements around may occur when the animal protects a painful limb and avoids putting weight on it. Thus, it is not surprising that the percentage of lying down movements with collisions were correlated positively with the percentage of lame cows (r_s_ = 0.45, P < 0.001), but also with the duration of lying down movements (r_s_ = 0.46, P < 0.001), the percentage of cows with at least one lesion (r_s_ = 0.33, P = 0.003), and the percentage of cows with mastitis (r_s_ = 0.38, P = 0.001).

Significant difference (t = -4.49, P < 0.001, df = 78) was found also for the cows resting partly outside the lying area, yet the situation was problematic only in the THSNE [13]. In the farms investigated by us the cows were lying partly outside the lying area probable due to the inappropriate dimensions of the bed (less than 170 cm long, less than 105 cm wide) and the absence of partitions between the stalls. This aspect was reported also by Mattiello et al. [11], who observed more abnormal lying down positions of the cows in tie-stalls with a length equal to or less than 175 cm. Lying partly outside of the bed can lead to additional soiling of the animals’ body and occurrence of skin lesions and infections. The percentage of cows resting partly outside the lying area correlated significantly with the duration of lying down movements (r_s_ = 0.64, P < 0.001), the percentage of cows with dirty lower legs (r_s_ = 0.24, P = 0.03), the percentage of lame cows (r_p_ = 0.50, P < 0.001), the percentage of cows with at least one hairless patch and no lesion (r_p_ = 0.49, P < 0.001), the percentage of cows with at least one lesion (r_p_ = 0.39, P < 0.001) and with the percentage of cows with dystocia (r_s_ = 0.42, P < 0.001). Impaired lying down behaviour (longer lying down durations, more collisions during lying down and more cows lying partly or completely outside the lying area) may result in decreased resting quality in general and can increase the risk for health problems such as body lesions or lameness as our study demonstrated it by the correlations found.

Both in THSE and in THSNE farms the poor hygiene (Table 2) represented a serious welfare problem [13]. There were no significant differences (P > 0.05) between the two housing types regarding the percentage of the cows dirty in the three body regions assessed. Similar to other studies [24-26], positive correlations were demonstrated between hygiene levels of the three body regions, namely udder and lower legs (r_s_ = 0.86, P < 0.001), udder and upper legs and flank (r_s_ = 0.75, P < 0.001) and also lower legs and upper legs and flank (r_s_ = 0.79, P < 0.001). In our study the upper leg and flank were considered dirty most frequently, followed by the region of the lower leg and the udder, respectively (Table 1). The obtained results are in agreement with the existing data in the literature, stating that cows kept in tie-stalls have higher hygiene scores in the body region of the upper leg and flank than the ones in free housing, because of lying down in the dejections laid in stalls [27]. This body region can also get soiled in poorly maintained stalls presenting elements splashed with dejections or through the movements of dirty tail around the hind section. In more than half of the investigated farms the manure removal was done manually, leading to deficient barn hygiene and vitiated air. The fact that barn cleanliness does not represent a priority for the farmers was highlighted also in other studies [15,25,27]. Poor hygiene of the three body regions demonstrated in this study is mainly caused by disregarding the recommendations for daily cleaning and bedding change in the barns but also by the improper stall length. Poor hygiene is a big problem in tie-stalls as the cow is both eating and lying in the same stall and the claws are often standing in manure. The exposure of the cows to dirt, mud and dejections constitutes the premise for increased percentage of sub clinical and clinical mastitis and lameness [28,29].

### Animal based measures of good health

It is widely accepted that the clinical signs associated with diseases and the incidence of health problems and body lesions are useful as welfare measures, because any disturbance of health means a decrease in welfare [30]. However, the absence of body lesions and diseases is not enough to prove adequate welfare of the animal. The animal based measures (mean, SEM, median, range) related to the principle of good health in tie-stall housing system with vs. without exercise are shown in Table 3. The health status of the animals in THSE was better than in THSNE farms (Table 3), which shows the positive influence of exercise on the cows’ health, as it was also concluded by Gustafson [7] and Regula et al. [9].

**Table 3:** **Animal based measures related to the principle of good health in tie**-**stall housing system with vs**. **without exercise**

**Measures**	**Tie**-**stall housing system with exercise**				**Tie**-**stall housing system without exercise**			
	(**n** = **40**)				(**n** = **40**)			
	**Mean**	**SEM**	**Median**	**Range**	**Mean**	**SEM**	**Median**	**Range**
% lame cows	15.12	0.67	15.15	13.88	22.21^***^	0.51	26.265	10.00
% cows with no lesion	89.13^***^	1.14	87.50	24.32	75.71	0.96	76.93	23.41
% cows with at least one hairless patch and no lesion	44.43	2.23	45.45	53.33	50.49^*^	1.50	51.02	36.79
% cows with at least one lesion	10.87	1.14	12.50	24.32	22.30^***^	1.28	22.86	30.28
Frequency of coughing per cow per 15 min	0.31	0.22	0.00	6.25	0.00	0.00	0.00	0.00
% cows with nasal discharge	0.16	0.11	0.00	3.12	0.41	0.28	0.00	8.16
% cows with ocular discharge	1.75	0.39	0.00	6.25	0.92	0.30	0.00	6.25
% cows with increased respiratory rate	0.47	0.33	0.00	9.37	1.29	0.51	0.00	11.54
% cows with diarrhoea	2.26	0.77	0.00	15.62	3.43	0.94	0.00	23.07
% cows with vulvar discharge	0.14	0.09	0.00	2.70	1.90	0.54	0.00	14.00
% mastitis	6.38	0.54	5.00	10.50	13.85^***^	0.78	14.50	19.00
% mortality during the last 12 months	0.80	0.19	0.00	4.00	2.40	0.21	2.28^***^	5.00
% dystocia	0.91	0.23	0.00	5.00	2.66	0.24	2.78^***^	5.76
% downer cows	0.23	0.09	0.00	2.00	0.69^*^	0.13	0.00	2.00
% dehorned cows	80.48	5.71	100.00	100.00	85.50	3.83	100.00	100.0

^*^ significant statistical differences at P< 0.05.

^***^ significant statistical differences at P< 0.001.

n = number of farms.

SEM = standard error of the mean.

The beneficial effect of the exercise on lameness reduction reported in the scientific literature (see below) was observed also in our study. The prevalence of lameness varied in the investigated farms from 8.57% to 30%, being significantly higher (t = -11.95, P < 0.001, df = 78) in the farms where the cows are tethered all the time comparing with those where the cows had outdoor access. Regula et al. [9] found a lameness prevalence of 21% (in 1999) and 17% (in 2000) in Swiss dairy cows kept in tie-stalls with minimal outdoor access during winter; in the same time in tie-stalls with regular outdoor exercise throughout the year the prevalence of lameness was lower. Also, Bielfeldt et al. [31] observed that lameness was more frequent in cows housed in tie-stall barns without exercise (13.2%) than in tie-stall barns with exercise (9.6%). Lameness represents a major welfare problem of the dairy cows worldwide. The increase in lameness prevalence is associated with solid concrete flooring [29], decreased resting periods in decubitus due to discomfort [32], uncomfortable and dirty barns [33], increased degree of dirtiness in cows hind legs [25,27] and the lack of biotin supplementation in lactating cows [34]. In our study a strong correlation was found between lameness and the duration of lying down movements (r_s_ = 0.79, P < 0.001). The percentage of lame cows also was positively correlated with the percentage of lying down movements with collisions (r_p_ = 0.46, P < 0.001), the percentage of cows lying partly outside the lying area (r_p_ = 0.50, P < 0.001), the percentage of cows with at least one hairless patch (r_p_ = 0.51, P < 0.001), the percentage of cows with at least one lesion (r_p_ = 0.63, P < 0.001), the percentage of dystocia (r_s_ = 0.65, P < 0.001) and the percentage of cows with vulvar discharge (r_s_ = 0.40, P < 0.001). Similar correlations were reported by Regula et al. [9] in Switzerland.

The proportion of cows with at least one hairless patch and no lesion (mild integument alterations) was statistically significantly lower (t = -2.26, P = 0.027, df = 68) in THSE than in THSNE (Table 3). The most affected body areas were the hock and the neck, probably because of the short beds, reduced quantity of bedding and the short chain used to tether the cows in some farms. In our study the number of cows with at least one hairless patch was higher than reported in other studies [15,35].

Lesions (percentage of animals with severe integument alterations – at least one lesion or swelling) were observed especially in the hock region, in the cows kept in THSNE, the difference between the two housing systems being significant (t = - 6.65, P < 0.001, df = 78). Other studies found also that the prevalence of hock lesions in cows kept in tie-stalls can be reduced by daily exercise of a constant duration [7] or by outdoor exercise for minimum 50 hours in a four-week period [36]. However, because the characteristics of indoor and outdoor housing systems vary greatly, the effects of outdoor access should be interpreted with care [37]. Hence, the causes of the body lesions in the farms investigated by us were most likely the short beds, the small amounts of bedding and the lack of exercise. We consider that for the cows tethered all year long even better housing conditions would be required than for those having outdoor access, exactly to compensate the lack of movement possibilities. Unfortunately we found that the situation is just converse: in many THSNE farms the beds were shorter (174.10 ± 3.14 cm) than in the THSE farms (197.25 ± 4.16 cm), less bedding was used and the tethering chain was shorter (60.25 ± 1.97 cm vs. 87.40 ± 1.41 cm). The inadequate length (too short) of the bed seems to be a more important factor in the development of body lesions than the bedding quantity, because we observed a higher frequency of body lesions in the cows in those farms with short beds and abundant bedding (4-5 kg/head/day) than in the farms with proper length beds and less bedding (1.5 kg/head/day or less).

The percentage of cows with diarrhoea (Table 3) recorded in THSNE farms draws attention because it exceeds the warning threshold [13]. Diarrhoea in dairy cows may have infectious, parasitical, metabolic, toxic or nutritional causes. As long as the milk production and the body condition of the animals is not significantly altered, the majority of the farmers usually neglect the diarrhoea episodes of their cows although these have negative effects on the cows health and can lead even to death by dehydration.

The number of the cows presenting vulvar discharge, even if significantly higher in THSNE farms (U = 550, P = 0.001), does not represent a welfare problem in the investigated farms [13]. The result is in agreement with the findings of Brunn et al. [38] who suggests that reproductive disorders were lower in grazing herds. They explained the result with the better musculature condition and health as a result of grazing.

The prevalence of mastitis (Table 3) was significantly higher (t = -7.86, P < 0.001, df = 78) in the THSNE farms than in THSE, but at an alarming level in both housing systems [13]. The positive effect of exercise in lowering mastitis incidence in tethered cows was also highlighted in other studies. Gustafson [7] noted that dairy cows tied continuously had more cases of subclinical mastitis than cows given the opportunity to exercise each day. Mastitis is considered to be one of the most frequent and costly diseases in the dairy industry [39]. Significant correlations were found between the percentage of mastitis and the percentage of cows with vulvar discharge (r_s_ = 0.48, P < 0.001) and the percentage of dystocia (r_s_ = 0.36, P = 0.001). Because in both housing system types the incidence of mastitis exceeded 4.5% an emergency action plan at farm level would be necessary [13].

Mortality was recorded at a significantly higher percentage in THSNE than in THSE (U = 502, P = 0.003), similarly to the results reported recently by Dechow et al. [40]. A recent study [41] showed that the risk of a cow dying was reduced between 46% and 75% in a grazing compared to a zero-grazing herd, depending on the milking system (automatic or traditional). Significant correlations were found between this measure and the percentage of cows with at least one lesion (r_s_ = 0.60, P < 0.001), the percentage of cows with increased respiratory rate (r_s_ = 0.45, P < 0.001), the percentage of cows with vulvar discharge (r_s_ = 0.40, P < 0.001), the percentage of mastitis (r_s_ = 0.63, P < 0.001), the percentage of dystocia (r_s_ = 0.45, P < 0.001) and the percentage of downer cows (r_p_ = 0.40, P < 0.001). Some of these relations are similar to the findings of other studies. For example, McConnel et al. [42] found that respiratory problems, lameness and high percentage of sick cows treated at least once with antibiotics are among the variables significantly associated with mortality level in dairy cows.

The frequency of dystocia was generally low in the investigated farms (Table 3) but significantly higher in THSNE than in THSE (U = 184, P < 0.001). Gustaffson [7] and Mee [43] state that the prevalence of dystocia can be increased by the lack of exercise. Bendixen et al. [44] showed that cattle on pasture have a reduced incidence of dystocia. Yet, this low frequency of dystocia may not be fully true because this information was communicated by the farmers and they generally tend to underestimate the problems in their farms.

In THSE farms fewer downer cows were found than in THSNE (U = 720, P = 0.041). The percentage of downer cows does not represent a welfare problem due to its low value recorded in both housing systems.

Other measures assessed within the criteria absence of diseases, principle good health (i.e. frequency of coughing, cows with nasal discharge, with ocular discharge, with increased respiratory rate) were not influenced by the housing system and had low incidence, without serious impairment to cow welfare (Table 3).

### Behavioural measures

Significant differences between the THSE and THSNE farms were obtained for all the behavioural measures (Table 4). The results indicate the positive effect of exercise on the behaviour of tethered cows. Veissier et al. [1] recommend that cows housed in tie-stalls be given regular access to an exercise area, because some effects of behavioural frustration are observed after only one day of tethering.

**Table 4:** **Behavioural measures in tie**-**stall housing system with vs**. **without exercise**

**Measures**	**Tie**-**stall housing system with exercise**				**Tie**-**stall housing system without exercise**			
	**(n = 40)**				**(n = 40)**			
	**Mean**	**SEM**	**Median**	**Range**	**Mean**	**SEM**	**Median**	**Range**
Frequency of butts per cow per hour	0.84	0.01	0.87^***^	0.34	0.52	0.05	0.64	0.87
Frequency of displacements per cow per hour	0.09	0.01	0.10	0.18	0.14	0.01	0.14^***^	0.12
% cows that can be touched	70.32^***^	3.01	76.91	67.08	48.79	2.76	50.45	59.39
% cows that can be approached by 50 cm but not touched	23.64	3.07	16.13	61.15	38.48	2.81	30.61^***^	49.21
% cows that can be approached between 50 cm and 1 m	4.97	1.34	0.00	24.49	8.24	1.32	8.00^*^	35.00
% cows that can’t be approached	1.06	0.35	0.00	6.12	4.39	0.96	2.04^*^	25.00
Qualitative behaviour assessment	-4.78^**^	0.21	-5.175	4.64	-5.50	0.17	-5.57	3.82

^*^ significant statistical differences at P< 0.05.

^**^ significant statistical differences at P< 0.01.

^***^ significant statistical differences at P< 0.001.

n = number of farms.

SEM = standard error of the mean.

Frequency of head butts and displacements was significantly higher in TSHE than in THSNE (P < 0.001) but lower than reported for loose housing systems [45]. An increased incidence of agonistic behaviours however may indicate unpleasant or stressful situations [46]. There were significant correlations between the indicators of agonistic behaviour and those related with the quality of housing (for example between the frequency of head butts and the duration of lying down movements r_s_ = 0.75, P < 0.001, the percentage of collisions with housing equipment during lying down r_s_ = 0.43, P < 0.001; between the frequency of displacement and percentage of cows lying partly outside the lying area r_s_ = 0.38, P < 0.001) and the measures of good health (for example between the percentage of head butts and the percentage of lame cows r_s_ = 0.71, P < 0.001, the percentage of cows with at least one lesion r_s_ = 0.42, P < 0.001 and the percentage of mastitis r_s_ = 0.44, P < 0.001).

The significantly higher number (t = 5.27, P < 0.001, df = 78) of the cows which can be touched observed in THSE can be considered another argument in favour of this system. Recent studies showed that the human-animal relationship is better in the tie-stall housing systems than in the loose housing system [11,47], and according to our results especially when the tethered cows have outdoor access. The significant differences found for the percentage of cows that can be approached by 50 cm but not touched (U = 396, P < 0.001), the percentage of cows that can be approached between 50 cm and 1 m (U = 568, P = 0.017) and the percentage of cows that cannot be approached (U = 563, P = 0.03) support the above statement.

The problem encountered within this study regarding the qualitative behaviour assessment (QBA) of the cows was the limitation of their spatial mobility and of their possibility to display natural (or even unnatural) behaviours by the tethering system. Even if the tethered cows are somewhat motionless, the descriptors show what the body language of the assessed animals suggests. The scores for the QBA were significantly higher (t = 2.67, P = 0.009, df = 78) in THSE than in THSNE even if in both systems the negative welfare measures prevailed. The explanation of this fact might be that the cows are tethered. Yet, those cows benefiting from exercise showed a more positive emotional state (see the scores for QBA, Table 4) than those kept constantly in the barns. Similar results were reported recently by Vučemilo et al. [12] after a research accomplished in Croatia. Significant negative correlations were demonstrated between QBA and many welfare measures related with housing and health. QBA correlated strongly with the percentage of the cows lying partly outside the lying area (r_s_ = -0.62, P < 0.001) and moderately with the duration of lying down movements (r_s_ = -0.42, P < 0.001), percentage of cows with dirty lower legs (r_s_ = -0.48, P < 0.001), percentage of cows with dirty udder (r_s_ = -0.35, P = 0.002) and the percentage of cows with at least one lesion (r_p_ = -0.31, P = 0.006). Low negative correlations were found between QBA and different health-related measures, for example with the percentage of lame cows (r_s_ = -0.29, P = 0.008), the percentage of cows with ocular discharge (r_p_ = -0.29, P = 0.009), the percentage of cows with vulvar discharge (r_s_ = -0.23, P = 0.04) and the percentage of mastitis (r_s_ = -0.28, P = 0.01). Although the correlations calculated on data collected on farms in practice not necessarily reflects a causal relationship, it seems that if the cows do not have appropriate comfort conditions to rest and/or have health problems, they are less likely to show a positive emotional state. In addition, QBA correlated positively with the clean drinking bowls (r_s_ = 0.39, P = 0.002). A possible explanation of this correlation would be that cattle prefer to drink from clean water sources rather than from contaminated ones, when they are allowed to choose [48].

### Welfare criterion and principle scores

The descriptive statistics of the scores for the 11 welfare criteria and the four welfare principles in THSE and THSNE are shown in Table 5.

**Table 5:** **Descriptive statistics for principle and criteria scores in tie**-**stall housing system with vs**. **without exercise**

**Principles and criteria**	**Tie-stall housing system with exercise**				**Tie-stall housing system without exercise**			
	**(n = 40)**				**(n = 40)**			
	**Mean**	**SEM**	**Median**	**Range**	**Mean**	**SEM**	**Median**	**Range**
I. Principle: Good feeding	48.14	3.18	52.90^*^	59.70	35.99	3.87	37.60	59.20
Criterion: APH	63.34	4.93	58.90	79.40	62.26	4.72	52.50	82.30
Criterion: APT	51.45^*^	3.26	60.00	57.00	37.20	5.10	60.00	57.00
II. Principle: Good housing	35.37	0.65	35.25^***^	12.80	18.67	0.55	18.30	13.40
Criterion: CAR	39.37	1.73	37.55^***^	34.60	25.65	1.41	24.70	36.60
Criterion: EM	34.00	0.00	34.00^***^	0.00	15.00	0.00	15.00	0.00
III. Principle: Good health	51.26	2.53	52.20^***^	59.40	32.87	1.46	31.85	36.60
Criterion: AI	63.77	1.99	63.40^***^	54.50	45.06	0.85	46.75	18.10
Criterion: AD	82.36	3.02	80.25^***^	69.80	60.34	3.39	65.55	58.80
Criterion: APIMP	40.05	4.52	41.00^***^	87.00	21.55	3.29	13.00	87.00
IV. Principle: Appropriate behaviour	41.76	0.89	41.35^***^	22.40	16.00	0.38	15.60	8.30
Criterion: ESB	99.66	0.16	100.00	3.40	100.00^*^	0.00	100.00	0.00
Criterion: EOB	72.73	0.65	72.60^***^	13.10	0.00	0.00	0.00	0.00
Criterion: GHAR	80.54	1.92	83.50^***^	37.70	65.78	2.20	65.90	58.80
Criterion: PES	15.85	1.26	13.05^*^	29.00	11.73	0.94	11.00	20.70

^*^ significant statistical differences at P< 0.05.

^***^ significant statistical differences at P< 0.001.

n = number of farms.

SEM = standard error of the mean.

*APH* Absence of prolonged hunger, *APT* Absence of prolonged thirst, *CAR* Comfort around resting, *EM* Ease of movement, *AI* Absence of injuries, *AD* Absence of diseases, *APIMP* Absence of pain induced by management procedures, *ESB* Expression of social behaviours, *EOB* Expression of other behaviours, *GHAR* Good human-animal relationship, *PES* Positive emotional state.

The scores for the principle of good feeding were significantly higher in THSE (U = 614, P = 0.047). Within this principle significant differences were recorded only for the criterion absence of prolonged thirst (APT). In many investigated farms the water provision for the animals was inappropriate quantitatively and qualitatively. In this system the majority of the participating farms had one water bowl per two cows and according to Andersson et al. [49] one water bowl per cow is recommended because the submissive cow drinks less and gives less milk than the dominant animal in every cow pair. Restricting the water intake of cows with 50% will result in 74% lower milk yield and more aggressive behaviour [50]. Furthermore, in 60% of the farms with both housing types, the water flow rates were low. Andersson et al. [49] stated that cows drink more if the water flow rate is high. Additionally, the drinkers were dirty in 80% of the THSNE farms and in 70% of those with THSE. Unfortunately, it seems that the Romanian farmers are still not aware of the importance of providing animals with unlimited access to clean drinking water.

The scores of the good housing principle but also for the criteria included, comfort around resting (CAR) and ease of movement (EM), were significantly higher (P < 0.001) in the farms allowing cows access to exercise (Table 5). In both housing systems low scores were given because of the cows’ being tethered. The permanent tethering of dairy cows is in contradiction with the requirements of animal welfare. EFSA [6] recommended that if tie-stalls are used, the management system should permit dairy cows the freedom to exercise and groom daily, except when climatic conditions do not permit them to do so.

The better health status of the cows in THSE was proved by significantly higher scores (U = 252, P < 0.001) than in THSNE (Table 5) for the principle good health and the included criteria. Given that in Romania shortening (or docking) of the cows tails is not practiced, the score for the criterion absence of pain induced by management procedures (APIMP) was influenced only by the dehorning practices of the cows. Certainly, the process of dehorning is a source of stress and pain in the animal, particularly if performed without anaesthesia or analgesia [51].

The difference was significant (U = 0.00, P < 0.001) between the two housing systems for the scores of the welfare principle appropriate behaviour, but also of the criteria included (Table 5). The assessment of social behaviours in cattle is very important as they are considered social animals, with complex communication channels and allelomimesis exhibited in many behaviours [52]. Yet, the high scores obtained in all of the assessed farms for the expression of social behaviours (ESB) criterion must be considered with extreme precaution. Permanently tied cows cannot display observable agonistic behaviours, as they are limited by the tethering chain. Additionally, in tie-stall conditions there is no possibility for the cows to develop social hierarchy as they do not have any interaction possibility with the other cows besides their close neighbours [52]. In the farms where the cows had access to an outdoor paddock, a minimal level of social interaction was observed between the animals. To establish if this is also one of the tethered housing’s effects more research would be needed.

For the expression of other behaviours (EOB) criterion higher scores were obtained in the farms where the cows have access to pasture than in the farms where the cows are permanently tethered in the barns (Table 5). The positive effect of grazing is recognized in the scientific literature. The access of the cows to pasture prevents and reduces the incidence of hock damage, lameness and claw disorders [3,53], improves the behavioural parameters [54], increases the resistance of the immune system [55], stimulates the reproductive function [56], thus increases the welfare degree of the cows. Finally, another pro-pasturing argument is the fact that the obviously positive mental state manifested by body language which can be observed in healthy mature dairy cows on a qualitatively and quantitatively adequate pasture can rarely be seen in stall conditions, no matter how appropriate the housings and their equipments are [57].

The lowest scores within the principle appropriate behaviour were obtained for the positive emotional state criterion, both in the THSE and THSNE farms. A main cause of this situation is probably the tethered housing system, which does not provide freedom of movement and limits the behaviour of the cows.

### Overall assessment

**Figure 1:**
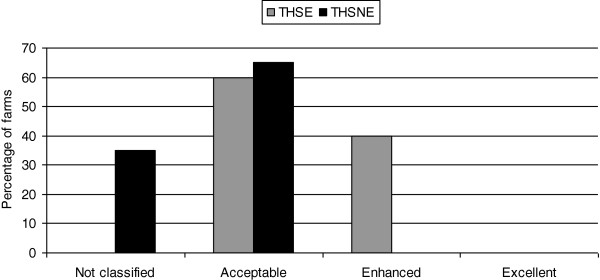
**Classification of the farms in different welfare categories based on the final score.** THSE = tie-stall housing system with access to exercise, THSNE = tie-stall housing system with no access to exercise.

The farms were classified in three of the four possible welfare categories based on the scores obtained for the four welfare principles: acceptable (24 THSE farms and 26 THSNE farms), enhanced (16 THSE farms) and not classified (14 THSNE farms) (Figure 1). No farm showed an excellent welfare level, similar to the results obtained in other countries [15,58]. Unfortunately, studies regarding the welfare assessment of the cows based on the Welfare Quality® protocol [13] in tie-stall systems had not been widely available until very recently, which made the comparison of the final results difficult. Ostojić-Andrić et al. [15] following a recent study accomplished in Serbia classifies the farms where the cows are kept tethered during the entire year in the acceptable (two farms) and enhanced (one farm) categories.

## Conclusions

The welfare quality of the investigated dairy cows was significantly better in the tie-stall farms which allow exercise for cows (paddocks, pasture or both) than in those which do not. In the light of our results we consider that dairy cattle welfare is not necessarily poor in tie-stall housing systems, its quality depending on the management practices. Tethered cows may have an improved welfare quality if they benefit from comfortable and clean stalls, quantitatively and qualitatively adequate feeding and watering, access to exercise and not in the least a good relationship with the stockperson. In this study the usefulness of the Welfare Quality® Assessment Protocol for Cattle [13] was proved for the first time in Romania, in spite of the high number of measures included. The welfare assessment based on the application of this protocol is utile especially because alongside the classification of the farms in a certain welfare category, it also helps to identify the positive and negative aspects at farm level. The implementation of adequate measures to correct the problems can significantly and efficiently improve the welfare of the animals.

## Abbreviations

THSE: Tie-stall housing system with access to exercise; THSNE: Tie-stall housing system without access to exercise; APH: Absence of prolonged hunger; APT: Absence of prolonged thirst; CAR: Comfort around resting; EM: Ease of movement; AI: Absence of injuries; AD: Absence of diseases; APIMP: Absence of pain induced by management procedures; ESB: Expression of social behaviours; EOB: Expression of other behaviours; GHAR: Good human-animal relationship; PES: Positive emotional state.

## Competing interests

The authors declare that they have no competing interests.

## Authors’ contributions

SP conducted the study and carried out the practical data collection at farms together with CB, EAD, MS, ISG and CDS. CB performed the measurements and calculations. SP performed the statistical analyses, and all authors contributed to interpretation of the data. SP and EAD drafted the manuscript. EAD realised also the English version of the text. ISG, MS and CDS revised the paper. All authors read and approved the final manuscript.
